# An Audit of the Use of a Ligature Assessment Tool in the Reporting of Ligature Incidents in a Regional Women’s Medium Secure Unit

**DOI:** 10.1192/bjo.2025.10688

**Published:** 2025-06-20

**Authors:** Thomas Teall, Bijal Arvind Sangoi, Zakaria Saidani

**Affiliations:** Birmingham and Solihull Mental Health Foundation Trust, Birmingham, United Kingdom

## Abstract

**Aims:** Ligature incidents in inpatient psychiatric settings represent a high-risk form of self-harm behaviour. Of all deaths that occur on psychiatric wards, 75% are caused by hanging or strangulation with a ligature. Accurate assessment and documentation of ligature incidents is essential for a comprehensive understanding of these complex incidents. A Ligature Assessment Tool was developed (Panchal et al, 2022) to improve and standardise the reporting of these incidents. Use of the tool provides a detailed source of information for the multi-disciplinary team, many of whom won’t have observed the incident first hand. These detailed reports can subsequently inform the care planning for individual patients.

The Ligature Assessment Tool was introduced in a regional women’s medium secure unit in 2020 and three audit cycles were completed assessing its use. This, the fourth and most recent audit, was conducted to further assess use of the tool.

**Methods:** Reports from the Eclipse incident reporting system, relating to ligature incidents between January and June 2024 (n=54), were reviewed retrospectively. Each report was reviewed and data was collected as to whether each of the Ligature Assessment Tool criteria were recorded as part of the written report. Data were collected as either “recorded” or “not recorded” for each of the 15 criteria of the Ligature Assessment Tool.

**Results:** There was a total of 54 reports relating to ligature incidents during the six month audit period. This audit showed further improvement in the use of the Ligature Assessment Tool, with 37% of reports recording 11 or more criteria from the tool, compared with 19% in the previous audit. 19 (35%) of the reports recorded all 15 criteria of the Ligature Assessment Tool.

**Conclusion:** This audit, conducted two and a half years after the previous audit, showed not only that the Ligature Assessment Tool continued to be used in the reporting of ligature incidents, but that the frequency of its use had increased. This occurred despite the fact that in the intervening period there had been no specific interventions, such as education or promotion, to improve use of the tool. This demonstrates its acceptability to staff and its ease of use, suggesting that tools such as this one could be integrated as part of normal practice in any setting. Going forward, the plan is to integrate the Ligature Assessment Tool into the incident reporting system within the Trust, meaning it will be used in all ligature incidents reports.

